# Diagnostic advancements in early detection of diabetic neuropathy: comparative analysis of medial and lateral plantar nerve degeneration

**DOI:** 10.1097/MS9.0000000000003441

**Published:** 2025-05-30

**Authors:** Ghazi Uddin Ahmed, Aliza Ahmed, Ahmed Asad Raza, Abdullah Bin Hameedullah, Syed Owais Akhtar, Amna Shakeel, Muhammad Bin Mobin, Abedin Samadi

**Affiliations:** a Department of Medicine, Jinnah Sindh Medical University, Karachi, Pakistan; b Department of Medicine, United Medical and Dental College, Karachi, Pakistan; c Department of Internal Medicine, Dow University of Health Science, Karachi, Pakistan; d Department of Medicine, Rashid Latif Medical College, Lahore, Pakistan; e Department of Medicine, Kabul University of Medical Sciences, Abu Ali Ibn Sina, Kabul, Afghanistan

**Keywords:** diabetic neuropathy, medial plantar nerve, lateral plantar nerve, early detection

## Abstract

Diabetic neuropathy (DN) is a widespread complication of diabetes, affecting nearly 50% of individuals with the condition. It commonly begins with the gradual loss of sensation in the lower extremities, particularly in the feet. If left undetected and untreated, this loss of sensation can lead to serious consequences, such as foot ulcers and eventual amputations. Early detection is critical to preventing irreversible nerve damage and minimizing the risk of such severe outcomes. However, current diagnostic methods often fail to identify neuropathy at an early stage, when intervention could still halt or reverse nerve degeneration. The medial and lateral plantar nerves, responsible for sensory innervation in the foot, are among the first to show signs of damage in DN. Monitoring these nerves offers a potential avenue for early diagnosis. Techniques such as nerve conduction studies (NCS) and quantitative sensory testing (QST) allow for the assessment of nerve function in the distal extremities, where neuropathy typically begins. Early identification of nerve dysfunction through these methods can lead to timely intervention, significantly improving patient outcomes and reducing the risk of severe complications. Addressing the early signs of neuropathy is essential in managing the growing prevalence of diabetes-related complications.

**HIGHLIGHTS**

- Early detection of diabetic neuropathy (DN) minimizes risks like foot ulcers and amputations.
- Medial and lateral plantar nerve degeneration are early indicators of neuropathy.
- Nerve conduction studies (NCS) are effective in identifying early diabetic nerve damage.
- Quantitative sensory testing aids in assessing sensory abnormalities in diabetics.
- Advancements in non-invasive diagnostic tools enhance early neuropathy detection.

**Table 1:** Divisions of the common digital nerves and their innervations

Divisions of the first common digital nerve	It supplies the adjacent aspects of the great and second toes^[7]^.
Divisions of the second common digital nerve	It supplies the adjacent aspects of the second and third toes^[7]^.
Divisions of the third common digital nerve	It supplies the adjacent sides of the third and fourth toes^[7]^.
	Moreover, the lateral plantar nerve provides a communication branch to the third common digital nerve^[7]^.

**Table 2:** Motor and sensory innervation of the medial plantar nerve

Motor innervation	It provides motor innervation to four of the plantar foot muscles: abductor hallucis, flexor digitorum brevis, flexor hallucis brevis and the first lumbrical^[7-9]^.
Sensory innervation	It provides sensory innervation to the majority of the skin on the medial two-thirds of the sole as well as the adjacent surfaces of the medial three and medial half of the fourth toe^[7-9]^.

**Table 3:** Innervation by the proper and common digital nerves of the lateral plantar nerve

The proper nerve	It supplies the lateral aspect of the little toe, the flexor digiti quinti brevis and the interosseous muscles of the fourth webspace^[23]^.
The common digital nerve	It communicates with the third common digital branch of the medial plantar nerve and divides into two digital nerves that supply the adjacent sides of the fourth and fifth toes^[24]^.

## Introduction

Half a billion of the people in the world are diabetics, which means that 10.5% of the adult population has this condition^[1]^. Due to its high prevalence, diabetes has led to a few associated disorders, of which the most common is the loss of sensation that starts distally in the lower extremities, known as diabetic neuropathy (DN). During their lifetime, almost 50% of diabetics develop this condition^[2]^, and because it causes a loss of sensation, DN can lead to substantial morbidity, foot ulcers, and in severe cases, the amputation of the foot^[3]^.

This review aims to address a critical gap in the early detection of DN. Despite its prevalence, there remains no widely accepted, reliable method for diagnosing the condition in its earliest stages before irreversible nerve damage occurs. By examining the degeneration of the medial and lateral plantar nerves, this review explores whether one nerve exhibits changes before the other and if this could serve as an early warning sign. Identifying such a pattern could help refine diagnostic techniques and improve patient outcomes.

The absence of a reliable and accurate test for the early detection of DN has led to it being diagnosed at later stages when irreversible nerve damage has already taken place^[4]^. Timely detection of DN can be compared to noticing a small crack in a bridge before it becomes a major structural issue. If detected in the early stages, the small nerve fibers in the foot can be protected from further damage, and to some extent, existing injury could be reversed. However, if caught during the late stages when loss of protective sensation of the foot has occurred, it is difficult to improve the condition and provide healthy treatment. Therefore, early detection and timely management would help reduce the incidence of ulcers and amputations, the prevalence of which has been increasing by 20-25% per annum among diabetics^[5]^.

For a better diagnosis, it is crucial to understand the anatomy of the nerves in the foot as the disease begins there. The sensory nerve supply of the foot is primarily supplied by the tibial nerve. At the level of the tarsal tunnel, the tibial nerve bifurcates into two nerves, the medial and lateral plantar nerves, just proximal to the medial malleolus. The medial plantar nerve, throughout its course, travels lateral to the posterior tibial artery, and after supplying the motor component, it provides sensory innervation to the medial side of the foot, which includes the first three digits along with the medial half of the fourth digit, whereas the lateral plantar nerve is responsible for the sensory innervation from the lateral side of the foot, including the lateral half of the fourth digit and the fifth digit^[6]^.

This review is focused specifically on the early detection of DN through changes in these two nerves. It does not cover other systemic complications of diabetes or peripheral neuropathies unrelated to diabetes. Additionally, while the discussion is centered on anatomical and functional degeneration, molecular or genetic factors are beyond its scope. The findings presented rely on existing literature, emphasizing the need for further clinical validation.

The medial and lateral plantar nerves can be imagined as two sentinel or vigilant guards that are responsible for keeping an eye out and guarding different areas of a palace, as both are responsible for taking sensory innervation from different structures in the foot. The objective of this review is to determine which of the two nerves is first to show degenerative changes and consequently loss of sensation in which side of the foot is more likely to happen first and serve as a potential early sign of DN, or in terms of the sentinel analogy, which of the two sentinels is first to raise the alarm?

### Methodology

#### Literature search strategy

A comprehensive literature search was conducted using databases such as PubMed, Scopus, Web of Science, and Google Scholar to identify relevant studies on early detection of DN, medial and lateral plantar nerve degeneration, and sensory nerve function in diabetic patients. The search was restricted to peer-reviewed articles, systematic reviews, and meta-analyses published in English from the past 20 years (2004–2024) to ensure up-to-date and relevant findings. Keywords and Medical Subject Headings (MeSH) terms included “diabetic neuropathy,” “plantar nerve degeneration,” “sensory loss in diabetes,” “early diagnosis of neuropathy,” and “foot ulcer prevention in diabetes.”

#### Inclusion and exclusion criteria

Inclusion criteria

- Studies involving human subjects diagnosed with diabetes.
- Research that specifically assesses medial and lateral plantar nerve function in diabetic patients.
- Studies employing clinical, electrophysiological, or imaging-based methods to evaluate neuropathy.
- Papers focusing on early-stage DN and its diagnostic approaches.

Exclusion criteria

- Studies focusing on neuropathies unrelated to diabetes (e.g., chemotherapy-induced neuropathy, hereditary neuropathies).
- Case reports, editorials, and opinion pieces without quantitative analysis.
- Research on animal models unless findings are directly translatable to human pathology.
- Articles published in languages other than English due to resource limitations in translation.

## Anatomy and physiology of the medial and lateral plantar nerves

As the tibial nerve descends distal to the tarsal tunnel and underneath or just distal to the inferior aspect of the flexor retinaculum, it terminates by splitting into the medial and lateral plantar nerves. These nerves enter the second layer of the foot and continue providing motor and sensory innervation to the plantar foot.

### The medial plantar nerve

#### Anatomical course

The medial plantar nerve, the larger and more anterior nerve amongst the two terminal branches, arises deep to the flexor retinaculum^[7-9]^. It crosses the posterior tibial artery on its lateral surface and runs anterior in relation to the lateral plantar nerve^[10]^. It is accompanied by the medial plantar artery along its course^[11]^. Continuing proximally, this nerve enters into the sole of the foot by passing between the quadratus plantae and abductor hallucis, reaching close to the master knot of Henry^[10]^. As it courses anterior to the abductor hallucis and flexor digitorum brevis, it innervates these muscles via motor branches on the lateral aspect of the medial plantar artery^[8]^. After innervating flexor hallucis brevis through motor branches and the first lumbrical, this nerve terminates near the bases of the metatarsal bones as sensory branches known as the three common plantar digital nerves that course laterally and the proper digital nerve of the great toe which travels in the medial compartment^[7,8,12,13]^. Table 1 summarizes the innervations of the three common digital nerves. The proper digital nerve of the great toe supplies the medial aspect of the foot including the great toe and the flexor halluces brevis muscle. The three common plantar digital nerves pass through plantar aponeurosis and each divide into two proper digital branches^[7,8]^. The first branch innervates the adjacent sides of the great and second toes; the second innervates adjoining sides of the second and third toes; whilst the third innervates the neighboring sides of the third and fourth toes and meets with the lateral plantar nerve. Each proper digital branch subsequently splits into cutaneous and articular divisions. Ultimately, the skin of the medial three-and-a-half toes including the dorsal skin, the associated nail beds, and some skin of the sole is supplied^[8]^. Table 2 sums up the motor and sensory innervation of the medial plantar nerve. It can be noted that the medial plantar nerve supplies more skin surfaces than musculature and is comparable to the median nerve of the upper limb in its distribution^[7,8]^.

As the medial plantar nerve travels deep to the flexor retinaculum, near the master knot of Henry, it may become compressed. This could result in a common condition among runners known as “jogger’s foot”^[14]^. This results due to repeated eversion of the foot. Paresthesia may develop at the navicular tuberosity and on the medial aspect of the sole. On MR imaging, the medial plantar nerve can be best seen with vasculature as it runs between the quadratus plantae and abductor hallucis muscles. On ultrasound imaging, the Henry master knot can be used as a landmark for identifying the nerve^[10]^.

#### Branches

The medial plantar nerve divides into articular, muscular, and cutaneous components^[15]^:

Cutaneous branches: After piercing the plantar aponeurosis between the abductor hallucis and the flexor digitorum brevis, the cutaneous branches are distributed along the sides of the medial three and the medial half of the fourth toe and extend over the dorsum of the foot^[7,9,16]^.

Muscular branches: The muscular branches innervate muscles located on the medial sole, such as the abductor hallucis, flexor digitorum brevis, flexor hallucis brevis, and the first lumbrical^[7,8,16]^. Furthermore, the nerves supplying abductor hallucis and flexor digitorum brevis arise from the main nerve trunk and travel to the deep muscle surfaces. In addition, the branch for the flexor hallucis brevis originates from the proper digital nerve (hallucal medial digital nerve), while the branch innervating the first lumbrical arises from the first common digital nerve.

Articular branches: The articular branches innervate the tarsus and metatarsus articulations^[7]^.

Proper digital nerve of the great toe: The proper digital nerve of the great toe innervates the skin on the medial aspect of the great toe and the flexor hallucis brevis muscle, lying next to the medial sesamoid bone^[13]^.

Three common digital nerves: The three common digital nerves traverse between the plantar aponeurosis and proceed towards the first, second, and third interdigital clefts. Each nerve divides into two proper digital nerves^[12]^.

Near the fifth phalanx, a dorsal branch moves upwards to supply structures surrounding the nail.

#### Functions

### The lateral plantar nerve

#### Anatomical course

The lateral plantar nerve originates below the flexor retinaculum and follows a more posterior course compared to the medial plantar nerve^[7,8,17]^. It travels deep to the proximal insertion of the abductor hallucis muscle and passes anterolaterally through the first and second layers of plantar muscles^[18]^. On its route, the lateral plantar artery accompanies this nerve^[11]^. Subsequently, it proceeds between the flexor digitorum brevis and quadratus plantae muscle, innervating them. Notably, the inferior calcaneal nerve, or Baxter’s nerve, stems from this nerve as it traverses the quadratus plantae muscle^[17,19]^. Arriving at the base of the fifth metatarsal, it splits into a deep and superficial branch^[9,20]^. The superficial branch further divides into two plantar digital nerves (a proper and a common) which go on to innervate the plantar skin of the lateral one and a half digits, dorsal skin, associated nail beds and some skin of the sole^[7,15,21,22]^. The deep branch travels between the third and fourth layers of muscles alongside the plantar arterial arch. This nerve supplies all muscles of the soles excluding those innervated by the medial plantar nerve and is analogous to the ulnar nerve of the upper limb^[7,8]^.

On MR imaging, the lateral plantar nerve can be seen more proximally between the flexor digitorum brevis and quadratus plantae muscles and more distally near the flexor digitorum brevis muscle^[10]^.

#### Branches

##### Superficial branch

The superficial branch splits into a proper and a common digital nerve^[15,17]^. The structures innervated by these branches have been outlined in Table 3.

##### Deep branch

The deep branch travels on the deep surface of the tendons of the flexor muscles and adductor hallucis. It is accompanied by the lateral plantar artery during its course. It supplies the remaining interossei, the second, third and fourth lumbricals, and adductor hallucis^[24]^.

#### Functions

**Figure 1.**
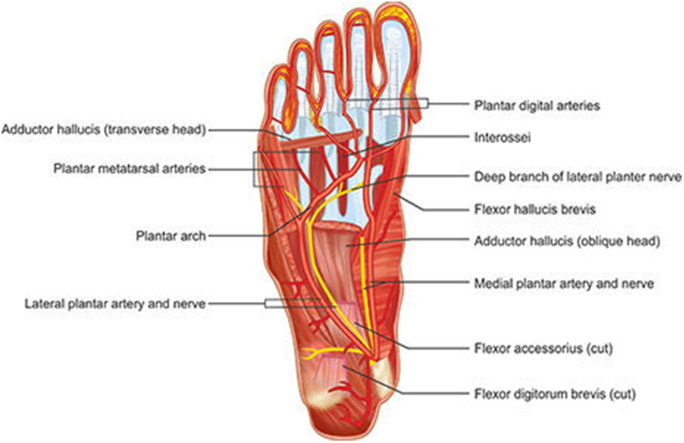
Anatomy and physiology of the medial and lateral plantar nerves^[25]^.

Table 4 describes the functions of these nerves meanwhile Fig. 1 depicts the anatomy.

**Table 4:** Motor and sensory innervation of the lateral plantar nerve

Motor innervation	Quadratus plantae and abductor digiti minimi via the main trunk^[7-9]^. Flexor digiti minimi brevis, interossei of fourth webspace space via the superficial branch^[7-9]^. Adductor hallucis, 2nd–4th lumbricals, interossei of the first three intermetatarsal spaces via the deep branch^[7-9]^.
Sensory innervation	The lateral plantar nerve provides sensory innervation to the lateral sole aspect and lateral one and one-half toes^[7-9]^.

## Diabetic neuropathy and plantar nerve involvement

Diabetes and prediabetes have become a global epidemic, consequently resulting in an epidemic of the complications of this disease, with one of the most common being diabetic peripheral neuropathy. It begins as a loss of sensory function distally in the lower limb, characterized by morbidity and pain^[2]^. The International Diabetes Federation estimates that 536.6 million people worldwide had diabetes in 2021, with the number predicted to increase to 783.2 million by 2045^[26]^. Almost half of diabetic individuals eventually go on to develop diabetic polyneuropathy (DPN)^[2]^. Pain induced by DPN affects the quality of life, making day to day life challenging. Distal symmetric polyneuropathy is the most common type of peripheral neuropathy in diabetic patients^[27]^.

The cell bodies of sensory neurons in the peripheral nervous system are located outside the blood-brain barrier, which makes them more vulnerable to diabetes-associated injury than motor neurons, whose cell bodies are located within the ventral horn and inside the blood-brain barrier, keeping them away from the systemic effects of diabetes^[28]^. Nociceptive information, particularly about heat and pain, are carried by C-fibers, which are small unmyelinated sensory neurons, making up most of the peripheral nervous system. Additionally, Aδ fibers, which are small but thinly myelinated, carry pressure, touch and cold sensations. For position and vibratory sensing, Aβ and Aα are responsible, which are fully myelinated large sensory fibers. Patients who develop pain, tingling, burning and prickling in their feet, have shown early damage to C and Aδ fibers. This is followed and accompanied by demyelination and then remyelination of the large fibers^[27]^.

The ends of the longest nerve fibers, particularly of the plantar, peroneal, and sural nerves are present in the lower extremities. Due to the length-dependent nature of DPN, its earliest manifestations hence appear in the lower extremities as well^[29]^. Due to its larger diameter, some authors concur that the earliest abnormalities involve the sural nerve, and hence, it is the most suitable nerve to study for early diagnosis of DPN^[30]^. Others suggest that studying the plantar nerve could also be suitable for early diagnosis, owing to it being more distal than the sural nerve^[31]^. Another aspect of the pathogenesis of DPN includes upregulation of inflammation and lipid processing pathways, observed in the nerves from preclinical models of type 1 diabetes mellitus (T1DM) and type 2 diabetes mellitus (T2DM). Furthermore, increased glucose levels lead to the activation of the polyol and hexosamine pathways, causing accumulation of reactive oxygen species, primarily due to mitochondrial injury, contributing to oxidative stress and ongoing neuropathy^[2]^.

Although the mechanisms differentiating neuropathy in T1DM and T2DM remain unclear, differences in progression suggest distinct influences. Neuropathy is more prevalent at diagnosis in T2DM, likely due to earlier metabolic and vascular dysfunction, whereas in T1DM, it develops later due to prolonged hyperglycemia. Glucose control has effectively shown to slow neuropathy in T1DM, with normoglycemia providing significant benefit, whereas T2DM neuropathy responds modestly to glycemic control, correlating more strongly with metabolic syndrome that accompanies it. Thus, early glucose optimization is key in T1DM, while a broader approach targeting multiple risk factors is essential in T2DM^[2]^. This highlights a significant research gap in understanding the distinct mechanisms, and hence neuropathy progression in the different types of diabetes.

## Diagnostic methods for plantar nerve involvement

Plantar nerve injury is diagnosed using a combination of imaging modalities, specialist tests, and clinical assessments, especially in situations such as DN or peripheral neuropathy^[32]^. The disease has subtle indications and symptoms, and the screening programs that are currently in use depend on subjective measures of major nerve fiber failure. Nerves function like cables, carrying, and conducting electrical impulses or signals between the brain and the body. One of the most crucial tools for diagnosing DN, which is considered the gold standard for the detection of neuropathy, includes the Nerve Conduction Study (NCS) parameters of the most distal sensory nerves of the lower extremities^[33]^. DN typically exhibits length-dependent changes in the distal nerves, primarily affecting the sensory nerves of the lower extremities. A NCS works by testing the transmission of these signals, especially the speed at which they travel and their “strength”^[34]^. The phenomena involve wire electrodes attached to the skin at specific positions along a nerve pathway. The nerve is stimulated by a healthcare professional via a mild electric shock, which travels down the nerve fiber in the form of an electric current. The speed and length of current are recorded by the electrodes. The reaction of the muscle that a motor nerve regulates is measured when it is stimulated. For the sensory nerve, the response is measured further along the nerve^[34]^. Slowed conduction velocity or reduced amplitude of the distal nerves proves that DPN is a length-dependent process, where the longest nerve fibers, which reach the toes, have the earliest anomalies. Thus, the assessment of distal nerve fiber conduction by stimulating them through electrical impulses can show evident signs of detection of neuropathy^[32]^.

Despite technological developments, clinical history and neurologic examination are critical in the diagnosis of many nervous system disorders. Another substantial way of detecting subclinical neuropathy in both symptomatic and asymptomatic patients is through quantitative sensory testing, a method to measure a person’s perception of different stimuli like vibration, thermal, pressure, etc. thresholds^[35]^. In individuals with confirmed neuropathy, identifying sensory anomalies by vibration perception threshold (VPT), pinprick, heat thresholds, and light touch testing has shown good specificity (77–96%), and positive predictive value (PPV) (95–98%) for DN ^[36]^. The occurrence of any level of impairment on evaluation examination, including any degree of weakness, reduced or absent reflexes, diminished or missing feeling in each sensory modality, decreased temperature sensation, and any gait anomaly, is considered abnormal, which is a consequent impairment in patients with DN. Approximately 50% of asymptomatic individuals with diabetes and normal NCS and over 93% of persons with impaired glucose tolerance (IGT) or type-2 diabetes (T2D) have abnormal heat sensitivity. A study discovered that patients with T2D had a higher heat detection threshold (HDT) and a lower cold detection threshold (CDT) of both the feet and hands than healthy volunteers^[37]^. Hence, by various quantitative sensory testing procedures, perception analysis of a patient can be done, allowing a comprehensive evaluation of plantar nerve damage often seen in such patients^[36]^.

A skin biopsy is another valuable diagnostic tool for assessing small fiber neuropathy, including plantar nerve damage in DN^[38]^. The primary goal of a skin biopsy in diagnosing neuropathy is to determine the density of the skin’s small nerve fibers. These tiny fibers, which are responsible for perceiving sensations like pain and warmth, may become damaged or smaller in number in DN. To diagnose small fiber neuropathy, a skin biopsy of the distal leg and thigh, considered the optimal sites, combined with intra-epidermal nerve fiber density (IENFD) measurement is regarded as the “gold standard”^[38]^. A significantly reduced IENFD, suggesting the degeneration of nerve fibers, indicates small fiber neuropathy, which is common in diabetes. Thus, unlike nerve conduction investigations, which focus on large nerve fibers, skin biopsy directly analyzes the small nerve fibers that are frequently the first to be impacted in DN. While minimally invasive, it is regarded as a highly sensitive and specific test for identifying small fiber neuropathy^[39]^. To offer a thorough assessment of neuropathy, a skin biopsy is usually done in conjunction with other diagnostic techniques, such as nerve conduction tests or quantitative sensory testing.

## Medial and lateral plantar nerve involvement as an early predictor of diabetic neuropathy

From a patient outcomes perspective, early intervention reduces the risk of serious complications, particularly foot ulcers, infections, and lower-limb amputations. Timely diagnosis can significantly alter clinical implications and improve patient outcomes. Detecting DPN in its early stages allows for early interventions, such as optimizing glycemic control and encouraging physical activity, which can slow or even prevent the progression of nerve damage. Early diagnosis also enables targeted treatment with neuropathy-specific therapies like alpha-lipoic acid or pregabalin before severe nerve impairment occurs.^[40]^ NCS of the sural nerve in the lower limb have been providing excellent results in the early detection of DPN in diabetic individuals and have been considered the gold standard. Patients with T2D show a highly significant decrease in the sensory nerve conduction velocity (NCV) of the sural nerve compared to non-diabetic individuals^[41]^. However, the involvement of the medial and lateral plantar nerves, both branches of the tibial nerve, has been a key point for researchers to further improve the early detection of DPN in diabetic patients.

Changes in the sensory latencies and amplitudes of the medial plantar nerve can be detected via NCS. These values can then be used as parameters to identify early signs of DPN in diabetic patients. In addition to the sural nerve, the medial plantar nerve has been successfully used for the early diagnosis of DPN in neurologically asymptomatic patients, as well as for confirming DPN in symptomatic patients^[42]^. On different occasions, the medial plantar nerve has provided better results than other nerves. The medial plantar sensory NCS proves to be a better diagnostic criterion for DPN in diabetic patients who have a routine NCS in the normal range compared to the sural nerve^[43]^. In addition to diagnosing more patients, NCS of the medial plantar nerve is also more accurate in detecting DPN than other peripheral nerves^[44]^. Hence, medial plantar NCS can be considered a better indicator of early signs of DPN.

There is a general dearth of research deducing the effects of NCS on the lateral plantar nerve separately. Comparatively, the medial plantar branch of the tibial nerve and its involvement in DPN has been researched adequately. This may be due to multiple reasons concerning the anatomy of both the lateral and medial plantar nerves, with the medial branch being the larger of the two and providing cutaneous supply to a larger part of the lower limb^[45,46]^. However, WPN conduction studies, which involve both the lateral and medial plantar nerves simultaneously, have started gaining importance recently. In patients suffering from type II diabetes mellitus, WPN conduction studies have emerged as a useful diagnostic tool for DPN. It is highly efficient as it detects NCV in the most distal sensory fibers of the lower limb. WPN conduction studies show high efficacy and accuracy in identifying early signs of DPN in patients with type II diabetes mellitus^[31]^.

Combining tests like NCS and Quantitative Sensory Testing (QST) can also prove fruitful in early diagnosis using parameters from the plantar nerves. If diabetic patients have a normal reading in NCS, combining NCS with QST might be a better early predictor^[38]^. The current literature does not provide sufficient evidence to definitively conclude whether the medial or lateral plantar nerve is more affected in DPN. Hence, it is not possible to state that studying one nerve over the other provides better efficacy in detecting early signs of DPN. In a study done by Kakrani *et al*, the involvement of the medial and lateral plantar nerves in patients with DN was found to be almost similar, with 76% of diabetic individuals having the involvement of both nerves,^[47]^ and Mondal *et al* supported this claim.^[48]^ This gap highlights a significant area for further research, as improving the early detection of DPN can enable early intervention to minimize further damage.

## Diagnostic and screening tools for DN

Diagnosing DPN in clinical settings usually entails evaluating the patient’s risk of developing a foot ulcer, frequently with the use of monofilament testing, and identifying whether the patient is symptomatic, particularly in the case of painful DPN. Nevertheless, DPN is only detected at a later stage by these techniques, and there are currently no easy indicators for routine early identification. The need for more standardized and early diagnostic methods is highlighted by the fact that while reliable biomarkers for monitoring DPN exist and are employed in clinical trials, existing clinical assessments are still imprecise, subjective, and largely dependent on examiners’ interpretations^[49]^.

There are several peripheral nervous system assessments available, each with advantages and disadvantages. The 10 g monofilament, the Ipswich Touch Test, and vibration perception threshold testing are common bedside tests used to diagnose DPN. These tests rely on the subjective responses of the patient and are primarily used to diagnose DPN at an advanced stage, with an emphasis on ulceration risk and loss of protective foot sensation. The effectiveness of early therapies that could stop DPN progression is limited by this delayed diagnosis, highlighting the significance of early detection and prompt treatment^[50]^. Table 5 summarizes the diagnostic tests available for assessing DPN.

**Table 5:** **Diagnostic tests available for assessing DPN**^[50-80].^

Test	Nerve fibers assessed	Advantages	Limitations
Symptoms and signs	Large (Aβ) and small (Aδ, C)	Easy to administer; useful for monitoring symptoms^[44]^.	Lacks sensitivity, accuracy,and reproducibility; subjective^[46]^.
NDS	Large (Aβ) and small (Aδ, C)	No need for specialized equipment; assesses large and small fiber function^[45]^.	No need for specialized equipment; assesses large and small fiber function^[45]^.
10 g Monofilament	Large (Aβ) fibers	Simple, quick, and cost-effective^[47]^.	Lacks standardized methods; not suitable for early neuropathy detection^[48]^.
Ipswich Touch Test	Large (Aβ) fibers	Simple, no special equipment needed; can be done at home^[47]^.	Only detects advanced neuropathy^[48]^.
QST (thermal and vibration thresholds)	Large (Aβ) and small (Aδ, C) fibers	Assesses large and small fiber function with good repeatability^[49,50]^.	Cannot differentiate peripheral from central abnormalities; high operator variability^[51,52]^.
DPN Check	Large, sural nerve (Aβ)	Quick, easy, with good sensitivity (92-95%) compared to NCS^[53,54]^	Limited to accessible sural nerve; validation studies had small sample sizes^[55]^.
NCS	Large (Aβ) fibers	Highly sensitive for assessing large nerve function; reproducible^[56,57]^.	Does not evaluate small fibers; is uncomfortable^[58]^.
Skin Biopsy (IENFD)	Small (C) fibers	The gold standard for small-fiber neuropathy; is quantitative; and detects early changes^[59,60]^.	Invasive, risk of infection, limited repeatability; requires trained personnel and specialized labs^[61]^.
CCM	Small (Aδ, C) fibers	Non-invasive with good reproducibility; rapid and objective^[61,62]^.	Expensive; requires specialized equipment and trained personnel; manual analysis is time-consuming^[63]^.
Neuropad	Small (C) fibers	Self-administered; suitable for screening; non-invasive with good sensitivity^[54,64-69]^	Interpretation of results can vary^[54,64-67]^.
Sudoscan	Small (C) fibers	Non-invasive, easy to perform^[70-72]^	Uncertain if it measures sudomotor function accurately; variable specificity (53-92%)^[70-72]^
QSART	Small (C) fibers	Sensitive for small fiber neuropathy^[73]^.	Time-consuming; requires specialized equipment and trained personnel; and is uncomfortable^[73]^.

IENFD: Intra-epidermal nerve fiber density; NCS: Nerve conduction studies; QSART: quantitative sudomotor axon reflex test; CCM: corneal confocal microscopy; NDS: neuropathy disability score; QST: quantitative sensory testing.

For early detection, Neuropad and Sudoscan provide non-invasive, rapid screening by assessing sudomotor function.^[61,71-79]^ Neuropad detects sweat gland dysfunction^[61]^, while Sudoscan evaluates electrochemical skin conductance, making them suitable for annual diabetes check-ups.^[78]^ However, their diagnostic accuracy varies, often requiring confirmatory tests. Corneal confocal microscopy (CCM) offers high-resolution imaging of corneal nerve fibers, an early marker of small fiber neuropathy, but its cost, specialized training requirement, and limited accessibility restrict routine use.^[68-70]^

Routine clinical screening primarily relies on bedside tests such as the 10 g monofilament test^[54,55]^ and Ipswich Touch Test^[54,55]^, which are cost-effective and easy to administer. These are recommended for annual foot exams to assess protective sensation loss, though they may not detect early neuropathic changes.^[50]^ The Neuropathy Disability Score (NDS) is another simple tool but lacks reproducibility due to its subjective nature.^[52]^

For confirmatory and advanced diagnostics, DPN Check, a point-of-care nerve conduction device, provides rapid sural nerve function assessment with high sensitivity. However, it only evaluates large fiber function and has been validated in limited studies.^[60-62]^ Traditional NCS remain the gold standard for large fiber neuropathy but are invasive, time-consuming, and require specialized personnel, making them more suitable for patients with atypical symptoms or suspected alternative diagnoses.^[63-65]^ Small fiber neuropathy confirmation relies on skin biopsy with IENFD analysis, the diagnostic gold standard for patients with neuropathic symptoms but normal NCS results. However, its invasiveness and histopathology requirements limit widespread use.^[66-68]^ Functional assessments such as QST^[56-59]^ and the Quantitative Sudomotor Axon Reflex Test (QSART)^[80]^ provide insight into small and large fiber integrity but require trained personnel and exhibit inter-operator variability.

The selection of diagnostic tests should be context dependent. In primary care, Neuropad, Sudoscan, and monofilament testing are viable for early detection.^[54,55,61,71-79]^ In specialist settings, confirmatory assessments such as CCM, QST, or IENFD should be employed for patients with unexplained neuropathic symptoms or normal routine test results.^[56,67,70]^ Research and specialized centers should reserve advanced techniques like QSART and NCS for detailed electrophysiological or structural evaluations.^[63,80]^

Despite the array of diagnostic tools available, significant challenges persist. Cost and accessibility limit the widespread use of advanced techniques like CCM and QSART^[68-70,80]^, while patient compliance can be affected by the invasiveness of procedures such as skin biopsy and the discomfort of NCS^[65]^. Moreover, diagnostic variability remains a concern, as tests like QST and Neuropad exhibit differences in sensitivity and specificity across populations.^[58,61]^ These barriers underscore the necessity for a structured, multimodal approach tailored to individual patient presentations and healthcare resource availability.

### *Discussion on the role of patient-reported outcomes (PROs) in the screening and diagnosis of* DN

According to the literature, 10–20% of patients who receive a diabetes diagnosis also receive a DPN diagnosis at the same time. However, studies that look at people who have had diabetes mellitus for a long time show that DPN is more common in those patients.

41% of diabetic people have neuropathy at 10 years, while 26% have peripheral neuropathy after 5 years. According to published research, between 50 and 66% of individuals with diabetes mellitus will experience DPN at some point in their lives.^[81]^ Both type 1 and type 2 diabetes can cause DPN, but because type 2 diabetes lasts longer and has more comorbidities, its prevalence is higher in those with the disease.^[82]^ With an incidence of 0.1–0.4% and up to 29% in patients with peripheral neuropathy, diabetes mellitus is also the most frequent cause of Charcot neuroarthropathy.^[83]^ Asymmetric sensory abnormalities can be clinically observed in approximately 50% of DPN patients^[84]^.

The risk of diabetes is increased by genetics and obesity. Among diabetes mellitus’s primary causes of morbidity are peripheral and autonomic neuropathies. At 5 years, the risk of mortality for diabetic foot ulcer patients is 2.5 times higher than the risk of death for diabetic foot ulcer patients without foot ulcers. Diabetic foot ulcers and related infections are more common causes of ED visits than congestive heart failure, renal illness, depression, and the majority of cancers.^[85]^

## Management and treatment

Managing DPN is difficult, particularly in T2D, where glycemic control alone frequently does not prevent DPN, in contrast to T1D.^[86]^ The American Diabetes Association (ADA) suggests that in T2D patients with DPN, glycemic control alone is insufficient^[49]^. Dietary weight loss has shown some promise; for instance, the Look Ahead study showed improved Michigan Neuropathy Screening Instrument (MNSI) questionnaire scores but no significant changes in clinical examination findings, indicating that weight loss may need to be combined with other early interventions for better outcomes.^[87]^ Exercise has also been shown to improve IENFD in T2D patients with DPN, independent of weight loss, indicating its potential benefit even without significant weight reduction.^[88]^

Since there are currently no efficient pharmaceutical interventions for DPN, sodium-glucose cotransporter-2 (SGLT-2) inhibitors present a potentially new therapy option, particularly in T1D, based on encouraging findings from animal research.^[89]^ Four main kinds of drugs are used to treat DPN: gabapentinoids, sodium channel blockers, tricyclic antidepressants, and serotonin-norepinephrine reuptake inhibitors. These medications have few side effects and are largely used to educate patients, take care of their feet, and decrease pain.^[90]^ Comprehensive, multimodal treatments are required to manage painful DPN, and non-pharmacological approaches including mindfulness and behavioral therapy also show early promise in this regard.^[91]^ Despite being prescribed occasionally, opioids are not recommended because of their long-term hazards and poor effectiveness.^[92]^ New studies on pain phenotyping could improve treatment personalization by focusing on certain pain pathways, which could lead to better outcomes^[93]^

A comprehensive review assessing non-pharmacological approaches to treating T2D in children and adolescents was discovered. Trials in this demographic are difficult to conduct, as evidenced by the fact that just seven research fulfilled the inclusion criteria. Diabetes education, calorie restriction, peer support, customised exercise regimens, self-management programs, and intensive lifestyle treatments were among the interventions that were studied.

Although the long-term consequences of very low-energy diets (VLEDs) are still unknown, they have been associated with weight loss and better glycemia. There are no randomized trials on bariatric surgery in young people, despite its high T2D remission rates. Some studies found that social support interventions had no effect on glycemia, while other investigations found inconsistent results. While individual views of parental participation were a factor, family-centered approaches seemed more promising. Programs run by medical professionals had varying effects on glycemia improvement.

To create efficient interventions that meet the particular social and psychological requirements of kids and teenagers with type 2 diabetes, more study is required.^[94]^

## Key findings and implications

This review emphasizes the differential involvement of the medial and lateral plantar nerves in DN, suggesting that one may undergo degenerative changes earlier than the other. Recognizing which nerve is affected first could provide a valuable early diagnostic marker, enabling timely interventions before significant nerve damage occurs. Early detection is crucial, as it allows for targeted management strategies, including neuroprotective therapies, lifestyle modifications, and sensory rehabilitation, potentially slowing disease progression and reducing the risk of severe complications such as foot ulcers and amputations.

Additionally, these findings highlight the need for standardized diagnostic criteria and objective, reproducible assessment methods to enhance early detection. Future longitudinal studies should further investigate the predictive value of plantar nerve involvement, improving risk stratification, and guiding clinical decision-making. By refining screening approaches, healthcare professionals can optimize patient outcomes, ultimately reducing the burden of DN on individuals and healthcare systems.

## Limitations and considerations

Despite its contributions, this review has certain limitations. The variability in study methodologies, including differences in sample sizes, diagnostic tools, and assessment techniques, introduces potential inconsistencies in the findings. Additionally, a significant challenge in synthesizing evidence is the potential for publication bias, as studies with statistically significant outcomes are more likely to be published, whereas negative or inconclusive findings may be underrepresented. Furthermore, the heterogeneity in study designs and diagnostic criteria may affect the comparability of results. To mitigate these limitations, this review has prioritized systematic reviews, meta-analyses, and large-scale cohort studies over small-sample studies that may lack statistical power. Nonetheless, caution should be exercised in generalizing the findings, and future research should aim for standardized methodologies to enhance reliability.

## Conclusion

By assessing the medial and lateral plantar nerves, we can detect and manage DN earlier, proving a critical step forward in decreasing the disease’s trajectory and remarkably lessening the burden of health infrastructure and shabby healthcare systems that are already stricken by many other pernicious diseases. The paramount importance can be highlighted by the fact that the medial plantar nerve has an extensive sensory distribution than many other nerves making it more susceptible to revealing signs of early neuropathic changes indicating an early detection of nerve damage being a valuable tool in identifying the onset of DN. On the other hand, the lateral plantar nerve, being less studied solely for the detection of DN, is of a significant diagnostic value when assessed alongside the medial plantar nerve through whole plantar NCS. Together, they offer crucial insights into the earliest stages of neuropathy, preventing this chronic condition from progressing, and creating severe complications such as foot ulcers and amputations.

The importance of this study cannot be overstated and calls for action to implement inexpensive diagnostic tests to evaluate and prognose diabetic neuropathies by screening the nerves. The NCS of the plantar nerve have already proven to be the best diagnostic method than the conventional methods which only detect neuropathy only after significant nerve damage. There should be screening centers set up for routine checkups for elderly patients who are more prone to diabetes and also there should be remote mobile camps set up to alleviate the disease in its latent stage. The healthcare providers should emphasize the dire neediness of this issue and raise concerns with the governmental organizations and NGOs to work with them to prevent severe morbidities in line with DN and the associated complications of nerve damage. Moreover, with the ongoing advancement in the field of biotechnology, it would be beneficial if some non-invasive nanotechnology prototypes were made to conduct NCS and monitor the progression of nerve damage in a diabetic patient all the time, integrating it into the health app and checking the blood glucose levels. This proactive strategy would be essential in benefiting the patients’ outcomes altering the course of the disease and revolutionizing the field of neuroscience.

Despite the complexities and challenges associated with managing DN, there is genuine hope for improvement. With greater awareness among healthcare professionals and patients, combined with the advancement of diagnostic techniques and treatment options, the outlook for those with diabetes can be significantly improved. Early and precise diagnosis not only facilitates more effective management but also empowers patients to take control of their health, maintaining a higher quality of life. This advancement in the evaluation of medial and lateral plantar nerves is a great stride in ensuring that people with a tendency to have DN have a healthier and more active lifestyle in the future.

Even though these diagnostic approaches show promise, further study is required to improve and broaden their therapeutic uses. Future research should look toward creating screening instruments for early neuropathy detection that are easier to use, less expensive, and non-invasive. Current treatment regimens may also be altered by longitudinal studies evaluating the efficacy of focused therapies based on early discoveries of nerve damage. Biotechnology developments, including as wearable biosensors and AI-powered diagnostics, have the potential to completely transform the diagnosis and treatment of neuropathy.

## Data Availability

No data is available.
